# Smartphone app for lifestyle improvement improves brain health and boosts the vitality and cognitive function of healthy middle‐aged adults

**DOI:** 10.1002/brb3.3500

**Published:** 2024-04-29

**Authors:** Keisuke Kokubun, Kiyotaka Nemoto, Yoshinori Yamakawa

**Affiliations:** ^1^ Open Innovation Institute Kyoto University Kyoto Japan; ^2^ Graduate School of Management Kyoto University Kyoto Japan; ^3^ Department of Psychiatry, Institute of Medicine University of Tsukuba Tsukuba Japan; ^4^ Institute of Innovative Research, Tokyo Institute of Technology Meguro Tokyo Japan; ^5^ ImPACT Program of Council for Science, Technology and Innovation (Cabinet Office, Government of Japan) Chiyoda Tokyo Japan; ^6^ Office for Academic and Industrial Innovation Kobe University Kobe Japan; ^7^ Brain Impact Kyoto Japan

**Keywords:** brain healthcare quotient, cognitive function, fractional anisotropy, smartphone apps, vigor

## Abstract

**Introduction:**

The number of smartphone apps for brain training is increasing, and the number of people who are working on brain training is also increasing. However, researchers disagree about the effectiveness of brain training.

**Methods:**

Therefore, in this study, we conducted an intervention test with the participation of 70 healthy middle‐aged men and women and measured the effect of smartphone apps on lifestyle improvement using brain healthcare quotient calculated from brain imaging data.

**Results:**

As a result, in the intervention group, significant improvements were seen in fractional anisotropy (FA) of the whole brain, corpus callosum, internal capsule, corona radiata, posterior thalamic radiation, external capsule, and superior longitudinal fasciculus. Additionally, in the intervention group, these FA increments correlated with improvements in cognitive function as measured by the trail‐making test and vigor as measured by the *Profile of Mood States 2nd Edition*.

**Conclusion:**

The results of this study suggest that improving lifestyle habits through smartphone apps can improve brain health and cognitive and emotional performance of healthy middle‐aged adults. This is consistent with previous research that suggests that FA integrity in the limbic‐thalamo‐cortical pathway influences cognitive function and emotion regulation.

## Graphical abstract

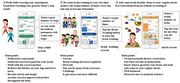

## 1 LIMITATION

This research has five limitations. First, there were significant differences in the initial values of FA and some regions between the intervention and control groups. The effect of the intervention was shown even in regions where there was no significant difference in the initial value, so it is considered that the effect of the difference in the initial value on the results was limited. Nonetheless, in the future, we should verify our results in participants with no significant difference in initial FA values between groups. Second, we did not give the control group any tasks. This may indicate that the psychological bias received from participation itself is not well controlled. Future studies should preferably verify the results of this study using active controls. Third, we did not give the intervention group any specific instructions other than to freely use the smartphone app. Additionally, we did not monitor or ask respondents about their app usage history in the post‐survey. Previous research has shown that not enjoying the use of apps and having privacy information obtained can be incentives for not using health‐related apps (Kim & Lee, 2022). However, this indicates that we may not confirm whether participants were taking part in the intervention nor control individual differences in frequency and content of use. Future studies should verify the results of this study by giving specific instructions on how to use smartphones or recording how the participants used the app. Fourth, the authors did not include the educational level of the participants in the test items. Therefore, it may not have been possible to exclude the influence of certain differences in intelligence among the participants on the results. Future research should confirm the robustness of the results of this study by including educational level as a test item. Fifth, in this study, the intervention period was short, 1 month, so, although the direct effects of the intervention were seen on FA, the effects on cognitive function and mood were only indirect. However, a recent 4‐month intervention study using the same app confirmed improvements in cognitive function in both diseased and healthy subjects (Kokubun, Toyama, et al., 2023). In the future, studies with longer intervention periods should be conducted to confirm the effect of the length of the intervention period on the results.

## 2 INTRODUCTION

Smartphone apps offering “brain exercises” aimed at enhancing cognitive function are proliferating (Meltzer et al., 2023). However, scientific evidence supporting the effectiveness of brain training in improving memory, general cognition, or daily functioning is lacking (Nguyen et al., 2021; Owen et al., 2010). There is consensus that brain training improves specific skills and leads to “near transfer” to related skills, whereas there is controversy about the potential for “far transfer,” where training‐developed skills enhance general cognitive resilience (Meltzer et al., 2023). Moreover, there is no clear standard for what is “near” and “far.” A recent intervention study by Nichols and others that combined a verbal inhibitory control task with a visuospatial working memory task found improvements in the trained task. However, it was shown that cognitively similar tests (near transfer) and cognitively different tests (far transfer) did not improve (Nichols et al., 2021).

The verbal inhibitory control training also resulted in changes in the left network of the frontotemporal and occipitofrontal tracts (involving the inferior longitudinal fascicle and the longitudinal occipitofrontal fascicle of the left hemisphere) of fractional anisotropy (FA), the most widely used diffusion tensor imaging (DTI) metric that can assess the integrity of white matter (WM) axonal tracts (Alexander et al., 2007). On the other hand, visuospatial working memory training resulted in changes in the right lateral frontoparietal tract (involving the superior longitudinal fasciculus and other WM tracts underlying the frontal and parietal regions in the right hemisphere). However, they found little overlap in changes between these training groups, suggesting that training results in changes in certain cognitive functions and brain regions, but not in other types of cognitive functions or brain regions (Alexander et al., 2007). The few changes common to both trainings occurred primarily in auditory, thalamic, and visual regions (Nichols et al., 2021). Other studies have measured the effects of brain training by changes in FA, but no consistent trends have been shown between studies regarding which brain regions have changed and how (Engvig et al., 2012; Sagi et al., 2012; Takeuchi et al., 2010).

Moreover, it has not been shown whether training results in the improvement of FA at the whole‐brain level. A study by Nichols et al. (2021) showed that not all changes in FA were in the same direction, and some regions showed decreases with training. A decrease in FA has been interpreted as reflecting a decrease in the overall directionality of the dome due to the increase in cross fibers, but the exact mechanism remains unknown. All we know is that there are regions where FA increases or decreases with training. These results suggest that brain training is expected to improve only specific brain regions and that it is difficult to expect improvements in FA at the whole‐brain level. Given that higher FA is generally thought to represent “better” WM integrity (Nichols et al., 2021), it is a non‐negligible problem that we do not know whether training will bring improvement in the whole brain even if it improves the brain and function in some areas.

Recent research examining the relationship between exercise and the brain shows that FA for the global WM was higher in middle‐aged healthy adults who had aerobic training for more than 10 years compared with that in sedentary adults of the same age group. Furthermore, the former had higher FA in the anterior, superior, and limbic WM tracts than the latter, including the genu of the corpus callosum, superior longitudinal fasciculus, and uncinate fasciculus (Tarumi et al., 2021). In addition, research investigating the relationship between diet and the brain includes a study showing that fish intake increases whole‐brain FA and cognitive function in healthy middle‐aged people (Kokubun, Nemoto, et al., 2020). These findings suggest that methods that combine brain training with guidance on improving various lifestyle habits are more effective in improving brain structure and cognitive function.

Even if brain training improves cognitive function, it will not be effective unless it motivates consumers to continue. That's why brain training needs to boost not just cognitive function, but happiness, energy, and motivation. Several researchers have had such an interest in the past. For example, a study of participants with executive dysfunction found that patients’ satisfaction and subjective well‐being improved after a combination of a multifaceted treatment program and a computerized brain training program (Spikman et al., 2010). In the same vein, a study of patients with acquired brain injury showed an improvement in their mood after computer training (Akerlund et al., 2013). These results may be due in part to the sense of accomplishment gained from completing the brain training task. Another possibility is that brain training, depending on the specifics of the program, is associated not only with cognitive enhancement but also with improved mood. In addition to the studies that the limbic‐thalamo‐cortical pathway regulates cognitive function (Catani et al., 2002; Kalivas & Volkow, 2005; Olivo et al., 2017), another study suggests that it plays an important role in regulating emotions (Sanjuan et al., 2013) and thus promotes happiness (Kokubun et al., 2022). However, to the best of the authors’ knowledge, there have been no studies demonstrating the relationship between brain training and FA in brain regions centering on the limbic‐thalamo‐cortical pathway.

Therefore, this research will verify the following hypotheses: (1) Brain training improves FA at the whole‐brain level; (2) changes in brain structure are accompanied by improvements in cognitive function and mood.

## 3 MATERIALS AND METHODS

The sample size required to perform a paired samples *t*‐test with a medium effect size *d* of.5, a significance level of 5%, and a power of 80% is calculated as 34 samples using G*Power 3.1.9.7. For comparison, the cognitive performance improvement of 2667 people who used the Lumosity Cognitive Training Program, which consists of 5 cognitive training tasks, for approximately 15 min, 5 days a week for 10 weeks, was *d* = .467 (Hardy et al., 2015). Therefore, it is reasonable to set *d* to.5 in this study, which deals with changes in brain structure and cognitive function. Considering the possibility that some participants would drop out during the study, 45 people were selected as the planned research participants for each of the intervention and control groups.

**FIGURE 1:**
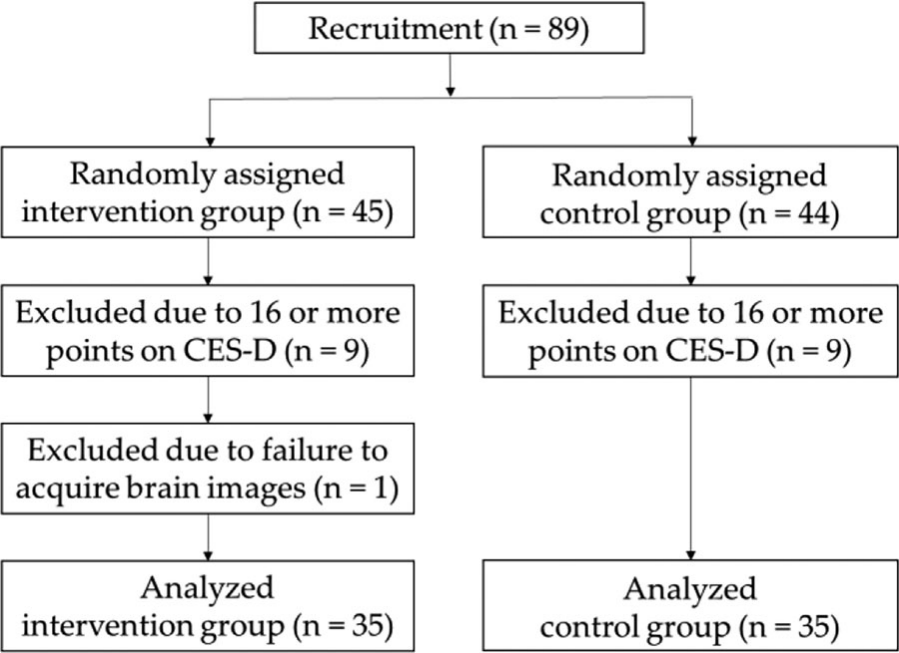
Flow diagram of the trial.

From October to December 2018, 89 healthy adults (47 females and 42 males) aged 40 to 68 years were recruited in Kyoto. According to self‐report, no participants recruited had records of neurological, psychiatric, or other medical conditions that could affect the central nervous system. Participants were randomly divided into intervention and control groups in a double‐blind manner using the random number generation function of the Microsoft Excel 2016 version. After recruitment, we administered the Center for Epidemiologic Studies Depression (CES‐D) scale (Radloff, 1977) to screen for depression. As a result, 18 participants were excluded from the study because they had a CES‐D score of 16 or higher and showed depressive tendencies. In addition, one person was excluded because brain imaging information could not be obtained. As a result, 70 people (39 women and 31 men) aged 40–68 years old were used in this study (Figure 1).

The intervention group performed training using the “App Good for Brain” (hereinafter referred to as the app) developed by bspr Inc. (2023) “freely” every day for 1 month. In other words, without specifying the number of days or hours required for training, participants were allowed to use the app as they wished, up to the day of the posttest. In our previous study, 20 healthy people and 15 people with the disease both used the app as they liked for 4 months, with no restrictions on days or hours, and showed improved scores on cognitive tests that reproduce the specifications announced by the Japanese National Police Agency in our previous study (Kokubun, Toyama, et al., 2023). The reason why there were no restrictions on how to use the app nor monitoring how participants used the app was to reduce the impact that the decline in enjoyment using the app had on the effectiveness of the app. Recent research has shown that the annoyance of using apps and the fear of having sensitive information obtained can reduce motivation to use health‐related apps (Kim & Lee, 2022).

**FIGURE 2:**
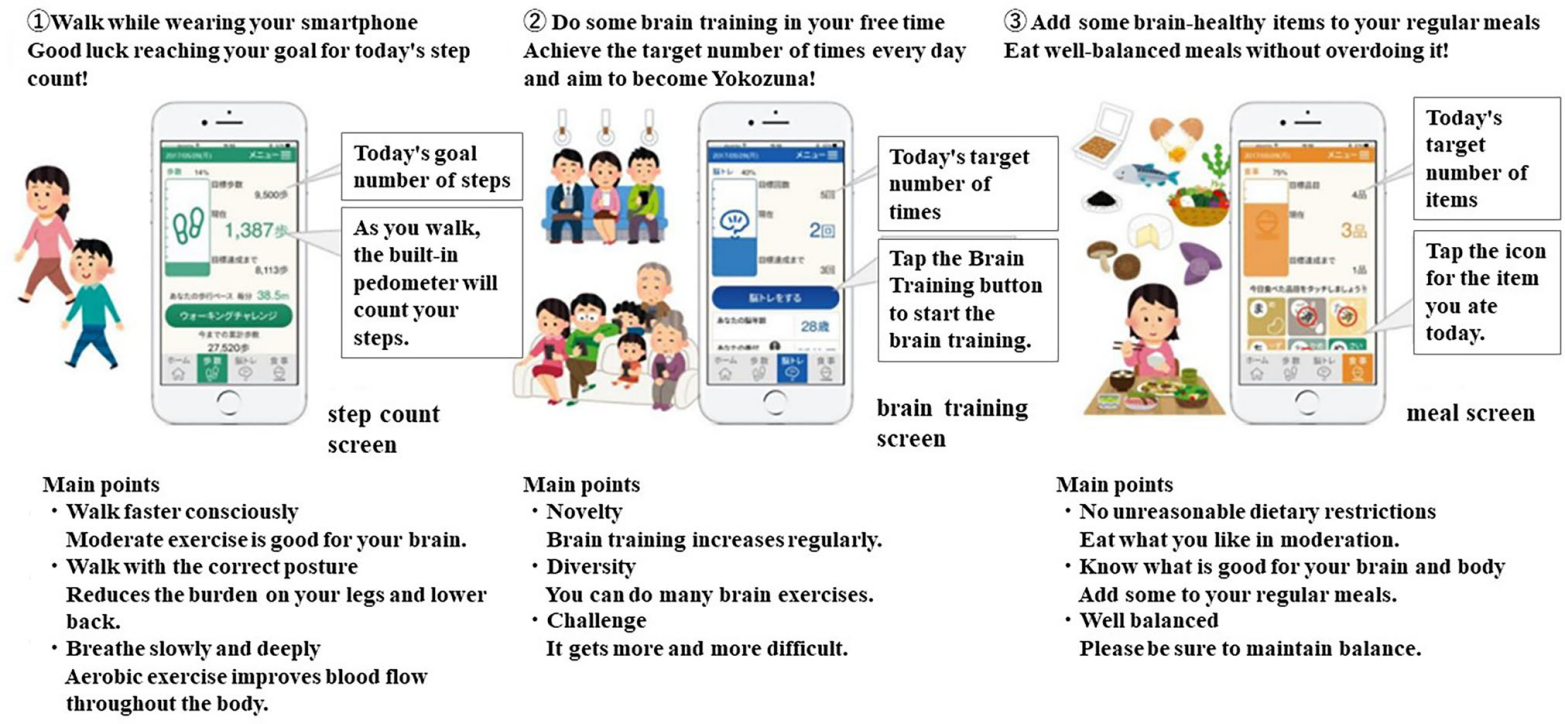
Characteristics of “App Good for Brain.” The authors translated Japanese materials provided by bspr Inc. into English.

This app has functions of notification and evaluation for “exercise,” “meal,” and “brain training” with artificial intelligence (AI), which learns the user activity and proposes the best for that person, designed to be “simple, fun, and easy to use” (from the bspr Inc. website). This app's brain training has a function that uses AI to change the difficulty level depending on the user's achievement level. The brain training consists of three types of brain training games: calculation quizzes, memory quizzes, and puzzle quizzes. Each session takes 3 min. Furthermore, these can be conducted in a competitive format between users. When training is complete, a radar graph of your performance and a “banzuke” (ranking list of sumo wrestlers) will be displayed. If you maintain good results, your ranking will go up; if you do not, your ranking will go down. In addition, the difficulty level of the quiz increases as the ranking increases. In addition, it has a function that counts the number of steps taken by the user to calculate how much exercise the user is lacking, calculates dietary imbalances from the user's food record, and then informs the user of these values (Figure 2). On the other hand, the control group did nothing during this period.

The primary and secondary outcomes of the study were changes in brain health as measured by brain healthcare quotient (BHQ), and changes in cognitive function and mood as measured by cognitive function and psychological tests, respectively. This study was approved by the Ethics Committee of Kyoto University (Approval Number 27‐P‐13) and was conducted following the institute's guidelines and regulations. All participants provided written informed consent before participation, and their anonymity was maintained.

### 3.1 MRI data acquisition

All magnetic resonance imaging (MRI) data were collected using a 3 Tesla MRI scanner (Verio, Siemens Medical Solutions or MAGNETOM Prisma, Siemens) with a 32‐channel head array coil. A high‐resolution structural image was acquired using a three‐dimensional *T*1‐weighted magnetization‐prepared, rapid‐acquisition gradient echo pulse sequence. The parameters were as follows: repetition time (TR), 1900 ms; echo time (TE), 2.52 ms; inversion time, 900 ms; flip angle, 9°; matrix size, 256 × 256; field of view (FOV), 256 mm; and slice thickness, 1 mm. DTI data were collected with spin‐echo echo‐planar imaging using generalized auto‐calibrating partially parallel acquisitions. Image slices were parallel to the orbitomeatal line. The parameters were as follows: TR =  14,100 ms; TE =  81 ms; flip angle =  90°; matrix size =  114 × 114; FOV =  224 mm; slice thickness =  2 mm. Baseline images (*b* = 0 s/mm^2^) and 30 different diffusion orientations were acquired with a *b*‐value of 1000 s/mm^2^.

### 3.2 MRI data analysis

*T*1‐weighted images were preprocessed and analyzed using Statistical Parametric Mapping 12 (SPM12; Wellcome Trust Center for Neuroimaging) running on MATLAB R2020b (Mathworks Inc.). Each MPRAGE image was split into gray matter (GM), WM, and cerebrospinal fluid (CSF) images using the SPM12 prior probability template. Subsequently, a diffeomorphic anatomical registration through an exponentiated lie algebra algorithm (Ashburner, 2007) was used to spatially normalize the segmented GM images. A modulation step was also incorporated into the preprocessing model to reflect the region volume and preserve the total GM volume before warping. As a final preprocessing step, we smoothed all segmented, modulated, and normalized images with an 8 mm full width at half‐maximum (FWHM) Gaussian kernel. The intracranial volume (ICV) was also calculated by summing the GM, WM, and CSF images for each participant. To control for differences in whole‐brain volume between participants, proportional GM images were generated by dividing the smoothed GM image by ICV. Mean and standard deviation (SD) images were generated from all participants using these proportional GM images. We then calculated the GM‐BHQ and defined the mean as BHQ 100 and SD as 15 BHQ points. By this definition, about 95% of the population is between BHQ70 and BHQ130. Individual GM quotient images were calculated using the following formula: 100 + 15 × (individual proportional GM − mean)/SD. Then, using an automated anatomical labeling atlas (Tzourio‐Mazoyer et al., 2002), regional GM quotients were extracted and averaged across regions to create a participant‐specific GM‐BHQ. In previous studies, whole‐brain GM‐BHQ was positively correlated with dietary balance (Kokubun & Yamakawa, 2019), curiosity (Kokubun, Yamakawa, et al., 2020), and behavioral activation (Kokubun, Yamakawa, et al., 2023). Moreover, whole‐brain GM‐BHQ was also negatively correlated with fatigue (Kokubun et al., 2018) and unhealthy lifestyle (Kokubun et al., 2021).

DTI data were preprocessed using FMRIB software library 6.0.2 (Jenkinson et al., 2012). First, in the initial b0 image, all diffuse images were aligned, and motion and distortion correction were performed by eddy correction. FA images were computed using a DTIFit according to these corrections. FA images were then spatially normalized to standard Montreal Neurological Institute (MNI) space using FLIRT and FNIRT (here, data were smoothed with a FWHM of 8 mm). Mean and SD images were generated from all FA images after spatial normalization. Then, individual FA quotient images were calculated using the formula 100 + 15 × (individual FA − mean)/SD. Finally, using the Johns Hopkins University (JHU) DTI‐based WM atlas (Mori et al., 2008), regional FA quotients were extracted and averaged across regions to yield a participant‐specific FA‐BHQ. For details, see Nemoto et al. (2017). In previous studies, whole‐brain FA‐BHQ was positively correlated with cognitive function (Kokubun, Nemoto, et al., 2020) and happiness (Kokubun et al., 2022) and negatively with anxiety (Pineda et al., 2021). Longitudinal studies have shown that olfactory training (Watanabe et al., 2023) enhances whole‐brain GM‐BHQ, and participation in human resource development training (Kokubun, Ogata, et al., 2020) enhances whole‐brain FA‐BHQ, respectively.

In addition to the entire brain, previous studies have clarified the relationship between structural connectivity of FA and cognition and emotional regulation. Therefore, we defined eight subregions of FA‐BHQ in terms of regions of interest (ROI). Previous studies have shown that FAs in some regions of the limbic‐thalamic‐cortical pathway, such as corona radiata, internal capsule, and cingulum, regulate reward and cognitive processes (Catani et al., 2002; Kalivas & Volkow, 2005; Olivo et al., 2017) and emotions (Lanius et al., 2001, 2003; Sanjuan et al., 2013; Schuff et al., 2011). Others have discussed that the following regions are more important for the processing of emotions, memory, and language: superior longitudinal fasciculus (Kamali et al., 2014; Mesulam, 1998; Petrides & Pandya, 2002; Schmahmann et al., 2008), corpus callosum (Konrad et al., 2012; Müller‐Oehring et al., 2009; Zhang et al., 2020), external capsule (Charlton et al., 2010; Mayo et al., 2019; Nolze‐Charron et al., 2020), or prefrontal cortex/uncinate fasciculus/amygdala pathway (Ayling et al., 2012; Gaffan & Wilson, 2008; Kim & Whalen, 2009; Von Der Heide et al., 2013). Therefore, in this study, we will analyze the changes caused by the intervention and the correlation with changes in cognitive function and mood in these eight areas selected from the perspective of ROI.

#### 3.2.1 Psychological test

An abbreviated version of the *Profile of Mood States 2nd Edition* (POMS 2) was used to measure the mood of participants. This is a psychological scale for measuring a wide range of mood states composed of 35 questions on a 5‐point scale to evaluate AH (anger‐hostility), CB (confusion‐bewilderment), DD (depression‐dejection), FI (fatigue‐inertia), TA (tension‐anxiety), VA (vigor‐activity), and F (friendship) (Heuchert & McNair, 2012). This scale was often used to measure the degree of mood disorders and fatigue associated with various diseases or their improvement with treatment or medication (Elbers et al., 2012; Higashikawa et al., 2020). Moreover, in previous studies, this scale was also used to measure mood in healthy adults (e.g., Kokubun et al., 2018).

#### 3.2.2 Cognitive test

For the measurement of cognitive function, we adopted the trail‐making test (TMT). TMT is a tool used to measure cognitive domains such as attention, working memory, spatial exploration, processing speed, ordering, mental flexibility, and visuomotor skills (Bowie & Harvey, 2006). It consists of Parts A and B. In Part A, participants connect a series of 25 numbers in numerical order. In Part B, the participant connects a series of 25 numbers and letters in numerical and alphabetical order, alternating between numbers and letters. We measured the total time to complete both Parts A and B. In this manuscript, these are referred to as “TMT A” and “TMT B.” It is said that “TMT A” is mainly related to processing speed and attention span, whereas “TMT B” is mainly related to working memory and set‐shifting ability (Sánchez‐Cubillo et al., 2009). Then we calculated the difference (Part B–Part A). This is referred to as “TMT B‐A” in this manuscript. “TMT B‐A” reflects cognitive flexibility. TMT can comprehensively measure broad attention, working memory, spatial exploration, processing speed, persistence, and impulsivity. TMT is widely used as an evaluation method for higher brain dysfunction due to traumatic brain injury, mild cognitive impairment and relatively mild dementia, and relatively pure executive dysfunction represented by prefrontal cortex damage. Moreover, TMT is also used to measure cognitive ability in healthy adults (e.g., Nemoto et al., 2022).

### 3.3 Data analysis

Differences in changes between the intervention group and the control group were tested using analysis of variance (ANOVA) interaction (Time × Group). Changes within groups were then tested using a paired samples *t*‐test. Furthermore, we conducted a *t*‐test with zero correlation as the null hypothesis for the correlation coefficients between changes in FA‐BHQ in the whole brain and each region and changes in POMS and TMT in the intervention group and control group, respectively. For these tests, SPSS/Amos 26.0 software (IBM Corporation Software Group) was used. In addition, Fisher's *Z*‐transformation was performed using online software developed by Lenhard and Lenhard (2014) to test differences between groups in the correlation coefficients between changes in FA‐BHQ in the whole brain and by region and changes in POMS and TMT. In all of the above tests, the significance level was set at 5% for two‐sided tests.

## 4 RESULTS

Table 1 lists the initial values for the baseline intervention and control groups. There was a significant difference at the 5% level in FA‐BHQ (*t* = 2.179, *p* = .033), posterior thalamic radiation (*t* = 2.182, *p* = .033), cingulum (*t* = 2.439, *p* = .017), superior longitudinal fasciculus (*t* = 2.532, *p* = .014), and uncinate fasciculus (*t* = 2.465, *p* = .016), with the intervention group lower than the control group in all the item scores. In addition to these, there was a significant difference at the 5% level in the cognitive test TMT B‐A (*t* = 2.199, *p* = .032). This value was higher in the intervention group than in the control group (i.e., the intervention group had lower cognitive function as measured by TMT than the control group).

**TABLE 1:** Comparison of baseline intervention group and control group.

	Intervention			Control				
	*Mean*	*SD*	*SEM*	*Mean*	*SD*	*SEM*	*t*	*p*
*Whole brain*								
FA‐BHQ	95.445	4.254	.719	97.476	3.507	.593	2.179	.033^*^
GM‐BHQ	96.276	7.614	1.287	96.983	7.120	1.203	.401	.690
*Regional brain*								
Corpus callosum	94.737	6.367	1.076	97.283	5.213	.881	1.831	.071
Internal capsule	94.156	4.628	.782	96.118	4.581	.774	1.783	.079
Corona radiata	94.770	6.435	1.088	96.784	5.217	.882	1.439	.155
Posterior thalamic radiation	96.142	6.408	1.083	99.217	5.330	.901	2.182	.033^*^
External capsule	96.164	5.701	.964	98.301	4.248	.718	1.778	.080
Cingulum	95.574	4.452	.752	98.038	3.986	.674	2.439	.017^*^
Superior longitudinal fasciculus	96.795	5.024	.849	99.521	3.918	.662	2.532	.014^*^
Uncinate fasciculus	92.886	6.462	1.092	96.352	5.236	.885	2.465	.016^*^
*Psychological test*								
POMS AH	2.890	2.529	.428	2.690	2.349	.397	.343	.733
POMS CB	2.400	2.172	.367	1.630	1.784	.301	1.624	.109
POMS DD	1.940	2.338	.395	1.140	1.801	.304	1.604	.114
POMS FI	2.830	2.269	.383	3.140	3.040	.514	.490	.626
POMS TA	3.510	2.801	.473	3.110	2.676	.452	.611	.543
POMS VA	10.690	4.745	.802	12.460	3.936	.665	1.700	.094
POMS F	11.570	4.111	.695	12.830	2.925	.494	1.474	.146
*Cognitive test*								
TMT A	22.640	5.880	.994	22.060	4.795	.811	.452	.653
TMT B	51.867	23.570	3.984	46.091	11.599	1.961	1.301	.199
TMT B‐A	31.641	17.726	2.996	24.031	10.244	1.732	2.199	.032^*^
*Demographic variables*								
Age	53.660	8.731	1.476	52.260	6.599	1.115	.757	.452
BMI	22.143	3.366	.569	23.240	3.551	.600	1.327	.189
	N	%		N	%		*χ* ^2^	*p*
Male	14	40.00%		17	48.60%		.521	.470
Female	21	60.00%		18	51.40%			

*Note*: *n* = 35 for intervention; *n* = 35 for control; ^*^ *p* < .05; ^**^ *p* < .01; ^***^ *p* < .001. No participants had depressive tendencies according to the results of the Center for Epidemiologic Studies Depression (CES‐D) scale (Radloff, 1977). In addition, no participants had records of neurological, psychiatric, or other medical conditions that could affect the central nervous system.

Abbreviations: AH, anger‐hostility; BMI, body mass index; CB, confusion‐bewilderment; DD, depression‐dejection; F, friendship; FA‐BHQ, fractional anisotropy brain healthcare quotient; FI, fatigue‐inertia; GM‐BHQ, gray matter brain healthcare quotient; POMS, Profile of Mood States; SD, standard deviation; TA, tension‐ anxiety; TMT A, trail‐making test Part A; TMT B, trail‐making test Part B; TMT B‐A, the score differences between TMT B and TMT A; VA, vigor‐activity.

**FIGURE 3:**
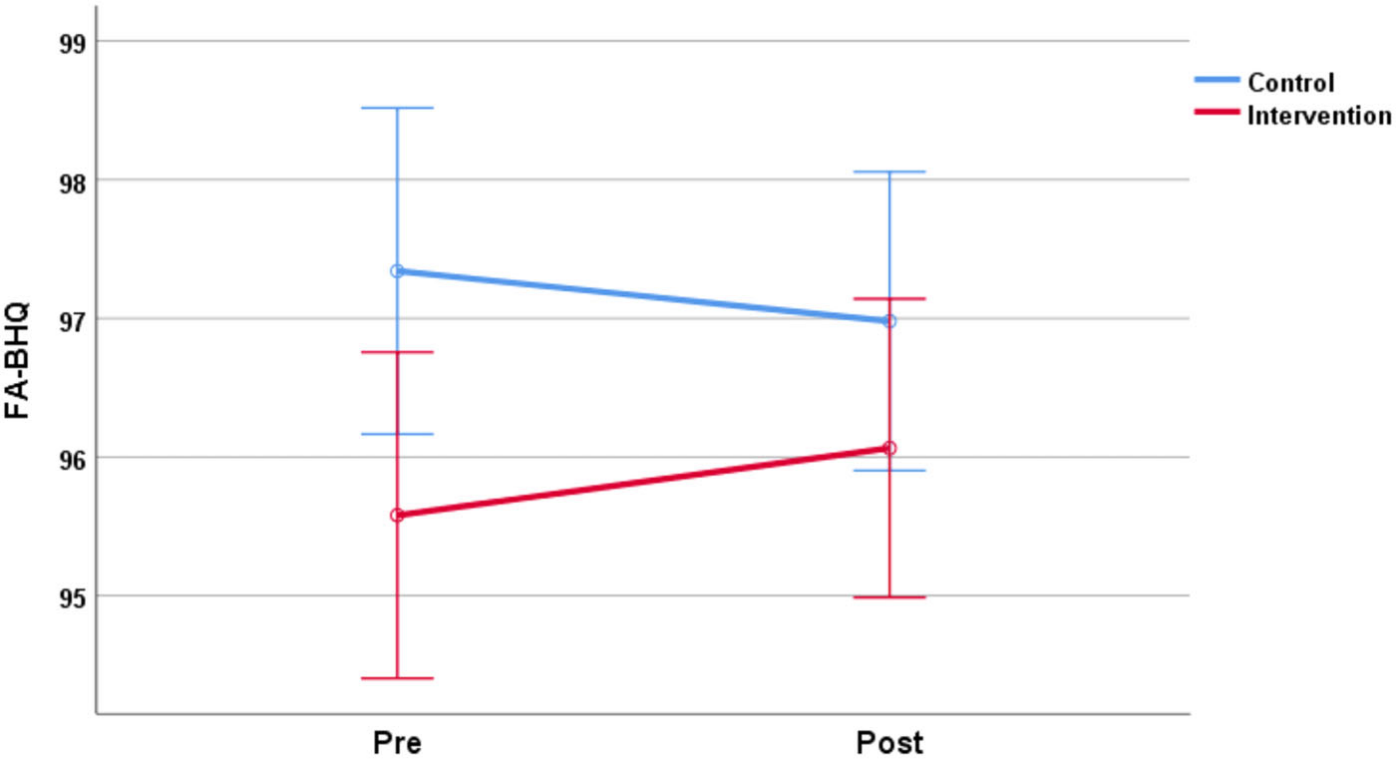
Fractional anisotropy‐brain healthcare quotient (FA‐BHQ) of the intervention group and control group before and after intervention by analysis of variance (ANOVA) method. Error bars are 95% confidence intervals.

Intervention effects were measured by a repeated measure ANOVA interaction (Time × Group). As a result, as shown in Table 2, there was a significant difference at the 5% level between the intervention group and the control group in an increase of FA‐BHQ (*F* = 5.292, *p* = .024, partial *η* ^2 ^= .072) (Figure 3). On the other hand, GM‐BHQ showed no significant difference (*F* = .387, *p* = .536, partial *η* ^2 ^= .006). In FA‐BHQ, by post hoc paired *t*‐test, there was a significant increase in the intervention group (*t* = 2.078, *p* = .045, *d* = .351), whereas no significant change in the control group (*t* = 1.281, *p* = .209, *d* = .217).

**TABLE 2:** Changes before and after the intervention (whole brain).

	Repeated measure ANOVA time × group			Paired *t*‐test intervention						Paired *t*‐test control					
	*F*	*p*	partial *η^2^*	Mean	SD	SEM	*t*	*p*	Cohen's *D*	Mean	SD	SEM	*t*	*p*	Cohen's *D*
FA‐BHQ pre				95.445	4.254	.719				97.476	3.507	.593			
FA‐BHQ post	5.292	.024^*^	.072	95.970	4.010	.678	−2.078	.045^*^	−.351	97.074	3.419	.578	1.281	.209	.217
GM‐BHQ pre				96.276	7.614	1.287				96.983	7.120	1.203			
GM‐BHQ post	.387	.536	.006	96.612	7.608	1.286	−1.218	.232	−.206	97.098	7.122	1.204	−.520	.607	−.088

*Note*: n = 35 for intervention; *n* = 35 for control; ^*^ *p* < .05; ^**^ *p* < .01; ^***^ *p* < .001.

Abbreviations: ANOVA, analysis of variance; FA‐BHQ, fractional anisotropy brain healthcare quotient; GM‐BHQ, gray matter brain healthcare quotient; post, data after the intervention; pre, data before the intervention; SD, standard deviation.

Looking at Table 3 by region, there was a significant difference in increase at the 5% level in ANOVA in the following regions: corpus callosum (ANOVA: *F* = 6.605, *p* = .012, partial *η* ^2^ = .089), internal capsule (ANOVA: *F* = 6.358, *p* = .014, partial *η* ^2^ = .086), corona radiata (ANOVA: *F* = 5.323, *p* = .024, partial *η* ^2^ = .073), posterior thalamic radiation (ANOVA: *F* = 5.091, *p* = .027, partial *η* ^2^ = .070), external capsule (ANOVA: *F* = 4.929, *p* = .030, partial *η* ^2^ = .068), superior longitudinal fasciculus (ANOVA: *F* = 5.963, *p* = .017, partial *η* ^2^ = .081). These variables were also statistically significant at the 5% level in multiple comparisons using the Benjamini and Hochberg method. On the other hand, there was no significant difference in increase at the 5% level for the cingulum (*F* = 3.796, *p* = .056, partial *η* ^2 ^= .053) and uncinate fasciculus (*F* = 2.905, *p* = .093, partial *η* ^2 ^= .041). Furthermore, looking at the results of the psychological test and cognitive test in Table 4, there was no significant difference in increase at the 5% level in ANOVA.

**TABLE 3:** Changes before and after the intervention (regional brain).

	Repeated measure ANOVA time × group			Paired *t*‐test intervention						Paired *t*‐test control					
	*F*	*p*	partial *η^2^*	Mean	SD	SEM	*t*	*p*	Cohen's *D*	Mean	SD	SEM	*t*	*p*	Cohen's *D*
Corpus callosum pre				94.737	6.367	1.076				97.283	5.213	.881			
Corpus callosum post	6.605	.012^*^	.089	95.301	5.994	1.013	−1.926	.062	−.326	96.705	5.293	.895	1.729	.093	.292
Internal capsule pre				94.156	4.628	.782				96.118	4.581	.774			
Internal capsule post	6.358	.014^*^	.086	94.980	4.492	.759	−2.278	.029^*^	−.385	95.530	4.200	.710	1.376	.178	.233
Corona radiata pre				94.770	6.435	1.088				96.784	5.217	.882			
Corona radiata post	5.323	.024^*^	.073	95.275	6.470	1.094	−1.836	.075	−.310	96.317	5.021	.849	1.463	.153	.247
Posterior thalamic radiation pre				96.142	6.408	1.083				99.217	5.330	.901			
Posterior thalamic radiation post	5.091	.027^*^	.070	96.718	6.295	1.064	−2.020	.051	−.341	98.836	5.451	.921	1.213	.234	.205
External capsule pre				96.164	5.701	.964				98.301	4.248	.718			
External capsule post	4.929	.030^*^	.068	96.918	5.383	.910	−2.556	.015^*^	−.432	97.990	3.898	.659	.823	.416	.139
Cingulum pre				95.574	4.452	.752				98.038	3.986	.674			
Cingulum post	3.796	.056	.053	95.976	4.130	.698	−1.440	.159	−.243	97.635	3.826	.647	1.323	.195	.224
Superior longitudinal fasciculus pre				96.795	5.024	.849				99.521	3.918	.662			
Superior longitudinal fasciculus post	5.963	.017^*^	.081	97.179	4.755	.804	−1.674	.103	−.283	98.970	3.519	.595	1.797	.081	.304
Uncinate fasciculus pre				92.886	6.462	1.092				96.352	5.236	.885			
Uncinate fasciculus post	2.905	.093	.041	93.577	6.531	1.104	−1.455	.155	−.246	95.946	5.445	.920	.935	.357	.158

*Note*: *n* = 35 for intervention; *n* = 35 for control; ^*^ *p* < .05; ^**^ *p* < .01; ^***^ *p* < .001.

Abbreviations: ANOVA, analysis of variance; post, data after the intervention.; pre, data before the intervention; SD, standard deviation.

**TABLE 4:** Changes before and after the intervention (psychological and cognitive test).

	Repeated measure ANOVA time × group			Paired *t*‐test intervention						Paired *t*‐test control					
	*F*	*p*	partial *η^2^*	Mean	SD	SEM	*t*	*p*	Cohen's *D*	Mean	SD	SEM	*t*	*p*	Cohen's *D*
Psychological test															
POMS AH pre				2.890	2.529	.428				2.690	2.349	.397			
POMS AH post	.108	.743	.002	2.830	1.871	.316	.132	.896	.022	2.800	2.506	.424	−.391	.698	−.066
POMS CB pre				2.400	2.172	.367				1.630	1.784	.301			
POMS CB post	.000	1.000	.000	2.400	1.850	.313	.000	1.000	.000	1.630	1.784	.301	.000	1.000	.000
POMS DD pre				1.940	2.338	.395				1.140	1.801	.304			
POMS DD post	.063	.803	.001	1.800	1.762	.298	.380	.706	.064	1.110	1.659	.280	.111	.912	.019
POMS FI pre				2.830	2.269	.383				3.140	3.040	.514			
POMS FI post	3.396	.070	.048	3.660	2.689	.455	−1.996	.054	−.337	3.090	2.716	.459	.236	.815	.040
POMS TA pre				3.510	2.801	.473				3.110	2.676	.452			
POMS TA post	.002	.962	.000	3.340	1.846	.312	.340	.736	.057	2.910	2.188	.370	.647	.522	.109
POMS VA pre				10.690	4.745	.802				12.460	3.936	.665			
POMS VA post	.920	.341	.013	11.140	4.930	.833	−.826	.415	−.140	12.260	3.995	.675	.496	.623	.084
POMS F pre				11.570	4.111	.695				12.830	2.925	.494			
POMS F post	.422	.518	.006	11.770	3.557	.601	−.486	.630	−.082	12.660	2.910	.492	.432	.668	.073
Cognitive test															
TMT Part A pre				22.640	5.880	.994				22.060	4.795	.811			
TMT PartA post	.099	.754	.001	19.957	4.550	.769	3.343	.002^**^	.565	19.757	5.531	.935	2.553	.015^*^	.432
TMT Part B pre				51.867	23.570	3.984				46.091	11.599	1.961			
TMT Part B post	.380	.540	.006	52.689	24.301	4.108	−.137	.892	−.023	43.131	13.973	2.362	2.325	.026^*^	.393
TMT B‐A pre				31.641	17.726	2.996				24.031	10.244	1.732			
TMT B‐A post	.107	.744	.002	32.731	24.381	4.121	−.226	.822	−.038	23.460	13.855	2.342	.359	.722	.061

*Note*: *n* = 35 for intervention; *n* = 35 for control; ^*^ *p* < .05; ^**^ *p* < .01; ^***^ *p* < .001.

Abbreviations: AH, anger‐hostility; ANOVA, analysis of variance; CB, confusion‐bewilderment; DD, depression‐dejection; F, friendship; FI, fatigue‐inertia; POMS, Profile of Mood States; post, data after the intervention.; pre, data before the intervention; SD, standard deviation; TA, tension‐ anxiety; TMT A, trail‐making test Part A; TMT B, trail‐making test Part B; TMT B‐A, the score differences between TMT B and TMT A; VA, vigor‐activity.

**FIGURE 4:**
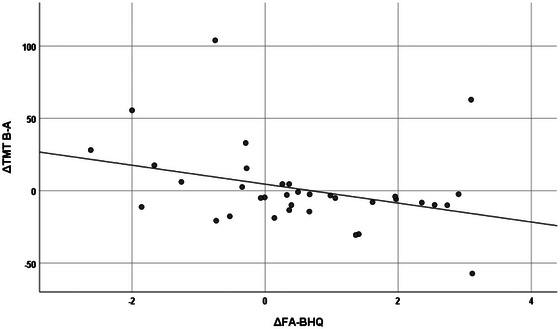
Correlation between fractional anisotropy‐brain healthcare quotient (FA‐BHQ) increments and trail‐making test (TMT) B‐A increments in the intervention group (*r* = −.343, *p* = .044).

**FIGURE 5:**
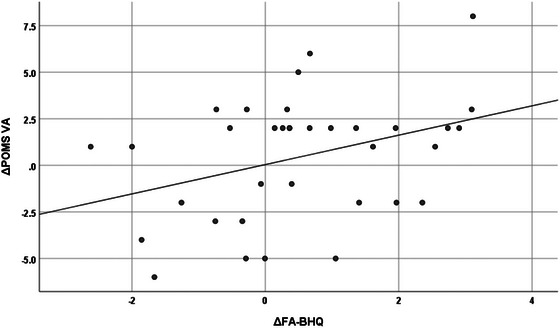
Correlation between fractional anisotropy‐brain healthcare quotient (FA‐BHQ) increments and Profile of Mood States (POMS) vigor‐activity (VA) increments in the intervention group (*r* = .359, *p* = .034)

**FIGURE 6:**
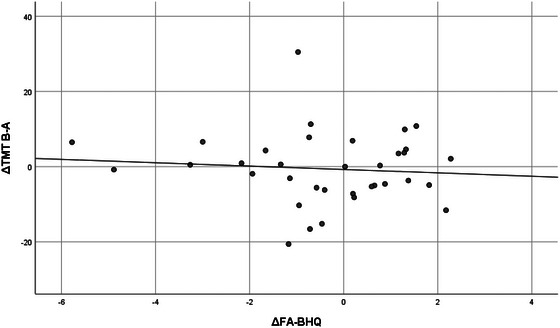
Correlation between fractional anisotropy‐brain healthcare quotient (FA‐BHQ) increments and trail‐making test (TMT) B‐A increments in the control group (*r* = −.089, *p* = .613).

**FIGURE 7:**
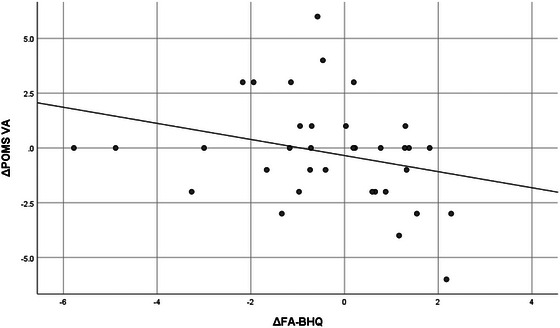
Correlation between fractional anisotropy‐brain healthcare quotient (FA‐BHQ) increments and Profile of Mood States (POMS) vigor‐activity (VA) increments in the control group (*r* = −.286, *p* = .096).

Table 5 shows the correlation between increases in whole FA‐BHQ and regional FA‐BHQ values and increases in psychological and cognitive test values, using data from the intervention group only. First, in the leftmost column, the increment of whole FA‐BHQ was significantly correlated at the 5% level with the increment of POMS VA (*r* = .359, *p* = .034) and the decrement of TMT B‐A (*r* = −.343, *p* = .044). Figures 4 and 5 are scatterplots showing these respective relationships. Similarly, the second and subsequent columns show correlations between regional FA‐BHQ increments and indicators. That is, the corpus callosum increment is associated with the TMT B‐A increment (*r* = −.366, *p* = .031); the internal capsule increment is associated with the POMS VA increment (*r* = .372, *p* = .028) and the TMT B‐A increment (*r* = −.399, *p* = .018); the increment of corona radiata is associated with the decrease of TMT B‐A (*r* = −.392, *p* = .020); and the increment of the external capsule is associated with the increment of POMS VA (*r* = .367, *p* = .030) and the decrease in TMT B‐A (*r* = −.438, *p* = .009), respectively. In addition, corpus callosum increment (*r* = −.335, *p* = .049), internal capsule increment (*r* = −.335, *p* = .049), corona radiata increment (*r* = −.335, *p* = .049), and external capsule increment (*r* = −.403, *p* = .016) were also correlated with a decrease in TMT B. On the other hand, as shown in Table 6 and Figures 6 and 7, no such relationship was observed in the control group.

**TABLE 5:** Change correlation in the intervention group.

	ΔFA‐BHQ		ΔCorpus callosum		ΔInternal capsule		ΔCorona radiata		ΔPosterior thalamic radiation		ΔExternal capsule		ΔSuperior longitudinal fasciculus	
	*r*	*p*	*r*	*p*	*r*	*p*	*r*	*p*	*r*	*p*	*r*	*p*	*r*	*p*
ΔPOMS AH	−.027	.875	−.044	.801	.002	.992	−.111	.527	.055	.754	.014	.935	−.070	.689
ΔPOMS CB	−.187	.283	−.198	.255	−.171	.327	−.254	.141	−.241	.163	−.122	.485	−.022	.900
ΔPOMS DD	.134	.443	.088	.617	.103	.555	.042	.812	.128	.463	.207	.232	.180	.301
ΔPOMS FI	−.177	.308	−.261	.130	−.130	.455	−.250	.147	−.186	.284	−.234	.177	−.282	.101
ΔPOMS TA	−.002	.992	−.022	.898	.013	.942	−.082	.639	−.006	.974	.076	.664	.079	.653
ΔPOMS VA	.359	.034^* (**)^	.268	.120	.372	.028^* (**)^	.280	.103	.292	.089	.367	.030^* (**)^	.313	.067
ΔPOMS F	.066	.706	.008	.964	.128	.465	.067	.701	−.082	.639	.090	.607	.133	.448
ΔTMT A	.101	.565	.122	.484	.156	.370	.099	.573	.093	.597	.131	.454	−.060	.732
ΔTMT B	−.304	.076	−.335	.049^*^	−.353	.038^* (*)^	−.376	.026^* (*)^	−.277	.108	−.403	.016^*^	−.230	.183
ΔTMT B‐A	−.343	.044^*^	−.366	.031^*^	−.399	.018*	−.392	.020*	−.299	.081	−.438	.009^**^	−.225	.195

*Note*: *n* = 35; ^*^ *p* < .05; ^**^ *p* < .01; ^***^ *p* < .001. The figures in parentheses are comparisons with the correlation coefficients in Table 6 using Fisher's *Z*‐transformation.

Abbreviations: AH, anger‐hostility; CB, confusion‐bewilderment; DD, depression‐dejection; F, friendship; FA‐BHQ, fractional anisotropy brain healthcare quotient; FI, fatigue‐inertia; POMS, Profile of Mood States; TA, tension‐ anxiety; TMT A, trail‐making test Part A; TMT B, trail‐making test Part B; TMT B‐A, the score differences between TMT B and TMT A; VA, vigor‐activity; Δ, change between before and after the intervention.

**TABLE 6:** Change correlation in the control group.

	ΔFA‐BHQ		ΔCorpus callosum		ΔInternal capsule		ΔCorona radiata		ΔPosterior thalamic radiation		ΔExternal capsule		ΔSuperior longitudinal fasciculus	
	*r*	*p*	*R*	*p*	*r*	*p*	*r*	*p*	*r*	*p*	*r*	*p*	*r*	*P*
ΔPOMS AH	−.009	.961	−.061	.729	.032	.854	.006	.973	−.026	.884	−.139	.424	.006	.971
ΔPOMS CB	−.081	.643	.000	1.000	−.116	.508	−.110	.528	−.103	.557	.019	.913	.035	.842
ΔPOMS DD	−.094	.590	.006	.971	−.124	.479	−.131	.453	−.145	.406	−.093	.596	−.136	.437
ΔPOMS FI	.008	.962	.079	.653	.024	.893	.021	.904	−.067	.703	−.075	.670	.010	.955
ΔPOMS TA	−.050	.777	.039	.825	−.107	.541	−.047	.787	−.176	.312	−.158	.366	−.140	.423
ΔPOMS VA	−.286	.096	−.327	.055	−.269	.118	−.272	.114	−.129	.461	−.230	.184	.015	.933
ΔPOMS F	−.168	.335	−.208	.231	−.180	.300	−.160	.359	−.067	.702	−.178	.307	−.019	.912
ΔTMT A	.239	.166	.219	.207	.232	.180	.284	.099	.109	.535	.181	.299	.268	.120
ΔTMT B	.064	.716	.062	.722	.058	.741	.142	.416	−.063	.719	−.046	.794	.021	.906
ΔTMT B‐A	−.089	.613	−.080	.647	−.087	.618	−.048	.784	−.114	.516	−.144	.410	−.137	.432

*Note*: *n* = 35; ^*^ *p* < .05; ^**^ *p* < .01; ^***^ *p* < .001.

Abbreviations: AH, anger‐hostility; CB, confusion‐bewilderment; DD, depression‐dejection; F, friendship; FA‐BHQ, fractional anisotropy brain healthcare quotient; FI, fatigue‐inertia; POMS, Profile of Mood States; TA, tension‐ anxiety; TMT A, trail‐making test Part A; TMT B, trail‐making test Part B; TMT B‐A, the score differences between TMT B and TMT A; VA, vigor‐activity; Δ, change between before and after the intervention.

Regarding the whole brain and regions where a significant correlation was observed in the intervention group, we compared the correlation coefficients between the intervention group (Table 5) and the control group (Table 6) using Fisher's *Z*‐transformation and found that in POMS VA increment, significant differences were observed in all of the cases: FA‐BHQ increment (*z* = 2.680, *p* = .004), internal capsule increment (*z* = 2.666, *p* = .004), and external capsule increment (*z* = 2.477, *p* = .007). In addition, significant differences were observed in some regions in TMT B increment: corpus callosum increment (*z* = −1.642, *p* = .05), internal capsule increment (*z* = −1.708, *p* = .044), corona radiata increment (*z* = −2.153, *p* = .016), and external capsule increment (*z* = −1.525, *p* = .064). On the other hand, in TMT B‐A, no significant differences were observed: FA‐BHQ increment (*z* = −1.073, *p* = .142), corpus callosum increment (*z* = −1.215, *p* = .112), internal capsule increment (*z* = −1.341, *p* = .09), corona radiata increment (*z* = −1.464, *p* = .072), and external capsule increment (*z* = −1.299, *p* = .097).

## 5 DISCUSSION

Intervention results showed that a smartphone app for brain training and lifestyle improvement significantly increased whole‐brain and regional FA, including corpus callosum, internal capsule, corona radiata, posterior thalamic radiation, external capsule, and superior longitudinal fasciculus. In addition, the intervention group showed the correlation of these whole and regional FA increments with the increases in cognitive function as measured by TMT and vigor as measured by POMS. On the contrary, no correlation was observed between these changes in the control group. Based on Fisher's *Z*‐transformation, a clear difference between the intervention group and the control group in the correlation coefficient of these changes was observed in vigor rather than TMT. In TMT, in some regions, a more significant between‐group difference in the correlation coefficient was observed in the change in test B than in the change in the difference between tests B and A. These are consistent with previous research that suggests that FA integrity in the limbic‐thalamo‐cortical pathway influences cognitive function and emotion regulation. However, in contrast to the whole‐brain FA and its subregions, the intervention did not result in a significant improvement in cingulum and uncinate fasciculus FA subregions and whole‐brain GM volume.

The posterior thalamic radiation, corona radiata, and internal capsule are all part of the limbic‐thalamic‐cortical circuit. The limbic‐thalamo‐cortical circuit plays a major role in emotional regulation (Drevets et al., 2008; Karababa et al., 2015; Li et al., 2023; Sanjuan et al., 2013; Yin et al., 2013). First, posterior thalamic radiation includes bidirectional thalamic cortical projections that connect the thalamic and basal ganglia, the visual, somatosensory, auditory, and gustatory cortices, and the prefrontal cortex. As such, it has been shown that abnormal WM integrity in posterior thalamic radiation can lead to emotional dysregulation (Kremkow & Alonso, 2018) and cognitive impairment (Zhu et al., 2015).

The corona radiata, on the other hand, are projection fibers that connect the thalamus and the cerebral cortex (Choi et al., 2015; Mori et al., 2008) and include thalamic projections from the internal capsule to the prefrontal cortex and parietal/occipital cortex (Catani et al., 2002). These pathways are associated with intellectual, social, emotional, executive, and attentional functions (Choi et al., 2015; Luders et al., 2007; Radoeva et al., 2012; Yin et al., 2013). As such, WM abnormalities in corona radiata can lead to cognitive and emotional dysregulation through the thalamus and internal capsule (Gaudio et al., 2017; Huang et al., 2022).

The internal capsule is part of the reward circuit along with thalamic radiation (Koch et al., 2014). The internal capsule, together with the corona radiata, is also part of a complex projection system that includes fibers from the thalamus to the cerebral cortex and from the frontoparietal cortex to the subcortical nuclei (basal and brainstem nuclei) and the spinal cord (Glickstein & Berlucchi, 2008). As such, the internal capsule is important for sensory, motor, and other higher cognitive functions (Frank et al., 2013; Olivo et al., 2017; Onoda et al., 2012).

In addition, the superior longitudinal fasciculus is a bundle of important associated fibers in the WM of each cerebral hemisphere connecting the parietal, occipital, temporal, and ipsilateral frontal lobes (Schmahmann et al., 2008). Therefore, superior longitudinal fasciculus is thought to facilitate the formation of bidirectional neural networks necessary for core processes such as attention, memory, emotion, and language (Kamali et al., 2014; Karlsgodt et al., 2008; Koshiyama et al., 2020; Mesulam, 1998; Petrides & Pandya, 2002). On the other hand, the corpus callosum connects the parietal, temporal, and occipital lobes (Fabri & Polonara, 2013). Therefore, previous studies have shown that corpus callosum is involved in emotion regulation (Konrad et al., 2012; Müller‐Oehring et al., 2009), and that the impairment of WM integrity in corpus callosum can lead to a lack of flexibility in cognitive behavior (Zhang et al., 2020). The external capsule is a corticostriatal fiber system that aids in the integration of information and carries fibers from the prefrontal, premotor, precentral, temporal, and preoccipital cortical regions to the forelimbs, putamen, and caudate nucleus (Schmahmann and Pandya 2006). Therefore, the external capsule is involved in working memory (Charlton et al., 2010; Nolze‐Charron et al., 2020) and executive functions (Mayo et al., 2019; Nolze‐Charron et al., 2020).

WM integrity in these regions, rather than individually, has been shown to contribute to some disorders. For example, FA reductions in both corona radiata and corpus callosum have been identified in multiple psychiatric disorders (Kelly et al., 2018; Riem et al., 2019; Thompson et al., 2016). Another study reported decreased FA for posterior thalamic radiation, corona radiata, and corpus callosum in patients with restrictive eating disorder (Laczkovics et al., 2022; Olivo et al., 2017). Therefore, the current research, which showed improvement in various regions centering on the limbic‐thalamo‐cortical pathway through the intervention, is positioned in the flow of such previous research.

However, some regions did not show any improvement effect. They are cingulum and uncinate fasciculus. Of these, the cingulum is a bundle of nerve fibers extending from the orbitofrontal cortex along the dorsal side of the corpus callosum to the temporal lobe and is part of the limbic system (Schmahmann et al., 2008). At the same time, the cingulum is one of the regions that compose the limbic‐thalamo‐cortical pathway together with corona radiata and internal capsule. Previous studies have shown that the cingulum is related to emotion regulation (Lanius et al., 2001, 2003; Sanjuan et al., 2013; Schuff et al., 2011) and affects cognitive function, including attention, memory, and motivation (Jang et al., 2019; Metzler‐Baddeley et al., 2012). On the other hand, the uncinate fasciculus, which connects the inferior frontal gyrus and the anterior temporal lobe region, also belongs to the limbic system and has been implicated in the processing of emotions, memory, and language (Ayling et al., 2012; Gaffan & Wilson, 2008; Kim & Whalen, 2009; Von Der Heide et al., 2013). In previous studies, FA values of cingulum (Jang et al., 2019) and uncinate fasciculus (Takahashi et al., 2002) were significantly lower in patients with cognitive impairment due to Alzheimer's disease and traumatic brain injury.

Therefore, it is unexpected that the cingulum and uncinate fasciculus did not change significantly with the intervention. Possible reasons include the method of screening participants. In this study, we excluded people with depressive tendencies from the research participants as it might be difficult for them to answer the questionnaire accurately and influence the results. However, multiple DTI studies dealing with depression have demonstrated reduced integrity in the cingulum (Bhatia et al., 2018; Bracht et al., 2015; LeWinn et al., 2014; Mincic, 2015) and uncinate fasciculus (Bhatia et al., 2018; Bracht et al., 2015; LeWinn et al., 2014). Therefore, future studies are required to investigate whether brain training affects FA in these areas and has the effect of improving mood and cognitive function in people with depressive tendencies.

Previous studies have not succeeded in showing scientific evidence that brain training improves cognitive function (Nguyen et al., 2021; Owen et al., 2010). Moreover, many previous studies have shown that brain training only leads to improvements in specific skills or “near transfer,” but not “far transfer” that involves improvements in general skills (Meltzer et al., 2023; Nichols et al., 2021). The same holds for research focusing on brain structures such as FA. That is, although previous studies have shown that various types of training improve specific regions of FA, there are no consistent results regarding which regions are improved and how (Alexander et al., 2007; Engvig et al., 2012; Sagi et al., 2012; Takeuchi et al., 2010). Furthermore, recent research has reported that brain training increases FA values in some areas, while decreasing them in others (Nichols et al., 2021). In light of previous research showing that FA is involved in emotion regulation (Sanjuan et al., 2013) and a sense of happiness (Kokubun et al., 2022) through the cooperation of multiple regions, it can be said that the effects of brain training have not been sufficiently proven yet. On the other hand, previous studies have shown that FA is related to exercise (Tarumi et al., 2021) and diet (Kokubun, Nemoto, et al., 2020), and that brain training is related to improving mood (Akerlund et al., 2013; Spikman et al., 2010). By combining brain training with the function of lifestyle improvement guidance, we can look forward to the development of a new app that can improve whole‐brain FA, cognitive function, and mood. Therefore, the fact that this study showed that the use of an app that combines brain training with diet and exercise improves whole‐brain FA and associated improvements in cognitive function and mood is important for future brain research and app development. This can be said to provide important hints.

Finally, because this study did not monitor participants’ app usage, it is difficult to directly demonstrate the relationship between usage and outcomes. However, according to our unpublished research, the apps used in the study tended to maintain overall adherence for at least 30 days, although the increase and decrease varied depending on the function (available upon request). This suggests that if the app has a variety of functions, even participants who would get bored with brain training alone will be less likely to get bored with exercise and eating, and as a result, it may be easier to achieve the desired outcomes.

The current study showed that an app developed for maintaining brain function by not only brain training but also improving exercise and diet improves whole‐brain and regional FA, including the limbic‐thalamo‐cortical pathway, which plays an important role in cognitive function and emotion regulation, accompanied by the improvement of cognitive function and mood, bridging the gap between recent brain training research and traditional brain‐related studies.

## 6 CONCLUSION

We conducted an intervention test with the participation of 70 healthy middle‐aged men and women and measured the effect of a smartphone app developed to maintain brain function by encouraging brain training, moderate exercise, and a well‐balanced diet using BHQ calculated from brain imaging data. As a result, in the intervention group, significant improvements were seen in FA of the whole brain, corpus callosum, internal capsule, corona radiata, posterior thalamic radiation, external capsule, and superior longitudinal fasciculus. Additionally, in the intervention group, these FA increments correlated with improvements in cognitive function as measured by TMT and vigor as measured by POMS. However, the intervention did not result in a significant improvement in cingulum and uncinate fasciculus FA subregions and whole‐brain GM volume. These are consistent with previous research that suggests that brain training alone cannot increase FA of the whole brain unless combined with moderate exercise and a well‐balanced diet and that FA integrity in the limbic‐thalamo‐cortical pathway influences cognitive function and emotion regulation.

## AUTHOR CONTRIBUTIONS

**Keisuke Kokubun**: Software; formal analysis; writing—original draft. **Kiyotaka Nemoto**: Conceptualization; methodology; writing—review and editing. **Yoshinori Yamakawa**: Conceptualization; data curation; funding acquisition; investigation; supervision; project administration; writing—review and editing.

## CONFLICT OF INTEREST STATEMENT

The authors have declared that no conflicts of interest exist. The bspr Inc. provided the app for use in the study but had no involvement in the design or conduct of the study. Additionally, neither the authors nor any organization to which they belong had any financial relationship with bspr Inc. at the time this intervention study was conducted.

### CONSENT FOR PUBLICATION

All participants gave consent for the publication of the results of this study.

### PEER REVIEW

The peer review history for this article is available at https://publons.com/publon/10.1002/brb3.3500.

## Data Availability

The datasets generated during the current study are not publicly available but are available from the corresponding author upon reasonable request.
